# *mcr-1*-Mediated Colistin Resistance and Genomic Characterization of Antimicrobial Resistance in ESBL-Producing *Salmonella* Infantis Strains from a Broiler Meat Production Chain in Italy

**DOI:** 10.3390/antibiotics11060728

**Published:** 2022-05-28

**Authors:** Patrizia Casagrande Proietti, Laura Musa, Valentina Stefanetti, Massimiliano Orsini, Valeria Toppi, Raffaella Branciari, Francesca Blasi, Chiara Francesca Magistrali, Stefano Capomaccio, Tana Shtylla Kika, Maria Pia Franciosini

**Affiliations:** 1 Department of Veterinary Medicine, University of Perugia, Via S. Costanzo 4, 06126 Perugia, Italy; laura.musa@studenti.unipg.it (L.M.); valentina.stefanetti@unipg.it (V.S.); valeria.toppi@studenti.unipg.it (V.T.); raffaella.branciari@unipg.it (R.B.); stefano.capomaccio@unipg.it (S.C.); maria.franciosini@unipg.it (M.P.F.); 2 Istituto Zooprofilattico Sperimentale delle Venezie, 35020 Padova, Italy; morsini@izsvenezie.it; 3 Istituto Zooprofilattico Sperimentale dell’Umbria e Delle Marche ‘Togo Rosati’, 06124 Perugia, Italy; f.blasi@izsum.it (F.B.); c.magistrali@izsum.it (C.F.M.); 4 Faculty of Veterinary Medicine, Agricultural University of Tirana, 1029 Tirana, Albania; tana.shtylla@ubt.edu.al

**Keywords:** colistin, *mcr* genes, *S*. Infantis, broiler chickens, antibiotic resistance, WGS

## Abstract

This work aimed to evaluate phenotypically and genotypically the colistin susceptibility of 85 *Salmonella* Infantis strains isolated in Italy from the broiler production chain, and to apply a whole-genome approach for the determination of genes conferring antimicrobial resistance (AMR). All isolates were tested by the broth microdilution method to evaluate the colistin minimum inhibitory concentrations (MICs). A multiplex PCR was performed in all isolates for the screening of *mcr-1*, *mcr-2*, *mcr-3 mcr-4*, *mcr-5* genes and whole-genome sequencing (WGS) of six *S*. Infantis was applied. Three out of 85 (3.5%) *S*. Infantis strains were colistin resistant (MIC values ranged from 4 to 8 mg/L) and *mcr-1* positive. The *mcr-1.1* and *mcr-1.2* variants located on the IncX4 plasmid were detected in three different colistin-resistant isolates. The two allelic variants showed identical sequences. All six isolates harbored *blaCTXM-1*, *aac(6′)-Iaa* and *gyrA/parC* genes, mediating, respectively, beta-lactam, aminoglycoside and quinolone resistance. The pESI-megaplasmid carrying *tet(A)* (tetracycline resistance), *dfrA1*, (trimethoprim resistance) *sul1*, (sulfonamide resistance) and *qacE* (quaternary ammonium resistance) genes was found in all isolates. To our knowledge, this is the first report of the *mcr-1.2* variant described in *S*. Infantis isolated from broilers chickens. Our results also showed a low prevalence of colistin- resistance, probably due to a reduction in colistin use in poultry. This might suggest an optimization of biosecurity control both on farms and in slaughterhouses.

## 1. Introduction

Colistin is a polypeptide antibiotic that was initially isolated in 1947 from the soil bacterium Paenibacillus polymyxa subsp. colistinus [1] and belongs to the group of polymyxins. In human medicine, colistin has been used for decades for the treatment of infections caused by Gram-negative bacteria and was later replaced by other antibiotics, such as aminoglycosides, quinolones and β-lactams, due to its toxicity [2]. Nowadays, the use of colistin is reconsidered as a last resort for infections caused by multidrug-resistant (MDR) bacteria [3]. In veterinary medicine, it has been used for a long time as preventive agent against Gram-negative infections [4] but also as a growth promoter in some other species of zootechnical interest [5,6]. The therapeutic usage of colistin is mostly related to enterobacterial infections, particularly in poultry and pigs with gastrointestinal infections, within intensive husbandry systems [7]. The World Health Organization has reclassified colistin in the category of drugs of “critical importance” for human medicine [8], justifying the execution of frequent studies addressed to monitor the resistance displayed by some bacteria, such as *Salmonella* spp. and *Escherichia coli*, largely widespread in both the human and veterinary field. *Salmonella* Infantis is considered an emerging serotype in Europe, widespread in broiler and turkey chain productions, configuring itself as the most common serotype after S. Enteridis and S. Typhimurium [9]. The progressive incidence of S. Infantis infections in humans was related to the spread of MDR S. Infantis strains along the broiler production chain carrying a pSEI-like plasmid with or without extended spectrum β-lactamase (ESBL)-production [10,11,12,13]. Recently, colistin resistance has been attributed to the mcr-1 gene (mobile colistin-resistant gene) located on a transferable plasmid, and first described in China [14] from animals, food and humans.

Nowadays, 32 *mcr-1* variants, 8 *mcr-2* variants, 40 *mcr-3* variants, 6 *mcr-4* variants and 4 *mcr-5*, are described in *Enterobacteriaceae* according to GenBank records (last accessed 23 December 2021). The predominant replicon types of plasmid-carrying *mcr-1* are IncI2, IncX4, IncHI2 and IncP [15]. The (Inc) plasmids groups are classified as incompatibility groups when two plasmids are unable to propagate steadily in the same host [16]. Up to now, 27 different plasmid incompatibility groups have been recognized in the *Enterobacteriaceae* family [17]. 

This work aimed to phenotypically and genotypically evaluate the colistin susceptibility of 85 *S*. Infantis strains isolated in Italy from the broiler production chain, and to determine the genes responsible for antimicrobial resistance (AMR) by whole-genome sequencing (WGS). 

## 2. Materials and Methods

### 2.1. Collection and Isolate Identification

Eighty-five *S*. Infantis strains collected in a previous study and selected for ESBL strain presence and for the phenotypic antibiotic resistance profile [18] were investigated. The strains were isolated from 2016 to 2017 in northern, central and southern Italy from cloacal samples (n = 13) on broiler farms and from environmental, skin, liver and meat by product samples (n = 72) from a slaughterhouse, following standard ISO procedures [19]. *Salmonella* spp. isolates were serotyped by direct slide agglutination with specific antisera (Statens Serum Institute, Copenhagen, Denmark), according to the Kaufmann–White–Le Minor scheme [20].

### 2.2. Colistin Susceptibility Testing

To assess colistin susceptibility, all *S*. Infantis isolates were analyzed by the broth microdilution method. Pure cultures were suspended in 4 mL of 0.90% sterile saline solution (final concentration: 5 × 10^7^ CFU/mL), equivalent to a 0.5 McFarland turbidity level (Vitek, bioMérieux Inc., Durham, NC, USA). Ten microlitres of bacterial suspension were transferred to 11 mL of cation-adjusted Müller Hinton broth (Thermo Fisher Scientific, Milan, Italy), and 50 μL of bacterial suspension were dispensed into each well of Euvsec FRCOL microtiter plates (Thermo Fisher Scientific, Milan, Italy) with scalar concentrations of colistin (COL, 0.12–128 mg/L). After inoculation, the plates were incubated for 24 h at 37 °C under aerobic conditions. The results were interpreted according to European Committee on Antimicrobial Susceptibility Testing (EUCAST) epidemiological cut-offs (MIC > 2 mg/L) [21]. *Escherichia coli* ATCC 25,922 and ZTA14/0097EC were used as quality and positive control strains, respectively.

### 2.3. Multiplex PCR Analysis for mcr Genes

Genomic DNA was extracted from individual colonies using the GenElute Bacterial Genomic DNA Kit (Sigma-Aldrich, Darmstadt, Germany) according to the manufacturer’s protocol. Extracted DNA quantity and quality were determined by spectrophotometry (NanoDrop, Thermo Fisher Scientific, Milan, Italy) and electrophoresis on 1% agarose gel, respectively. All isolates were screened by a multiplex PCR for *mcr-1*, *mcr-2*, *mcr-3 mcr-4*, *mcr-5* genes, as previously described [22]. One nanogram of DNA was used in a total of 50 µL of reaction mixture contained 10 × buffer, 3 mM MgCl_2_, 200 µM of each deoxyribonucleotide triphosphate, 1 µM of each primer (Sigma Aldrich, Milan. Italy), 0.5 U Taq DNA polymerase (Microtech, Padova, Italy). In each set of reactions, positive controls [22] and a negative control (negative sample), as well as a negative reaction mix control (containing the reagents and water instead of DNA), were included.

### 2.4. Whole-Genome Sequencing

Genomic DNA of six *S*. Infantis (3 *mcr-1* positive and 3 *mcr-1* negative) isolates was used for library preparation with a commercial kit (Nextera XT, Illumina San Diego, CA, USA). Libraries were sequenced using paired-end Illumina MiSeq, and the quality raw reads was checked using FastQC, (http://www.bioinformatics.babraham.ac.uk/projects/fastqc/) (accesse on 15 November 2021) removing those showing low quality (Phred Score < 20) and shorter than 70 nucleotides. Processed reads were de novo assembled using SPAdes, version 3.14 [23]. and the generated contigs were passed to CSAR v. 1.1.1 to build the scaffolds with more than 200 nucleotides in length. Subsequently, the genome assembly quality check was performed with QUAST v. 4.3, [24] and the sequences annotated using Prokka v. 1.12 [25]. Genome sequences were analyzed for the presence of genes encoding virulence factors, using databases. Genetic determinants of antibiotic resistance and plasmid replicons were assessed using “The Comprehensive Antibiotic Resistance Database” (CARD, https://card.mcmaster.ca/) (accesse on 15 November 2021) and the STARAMR software (https://github.com/phac-nml/staramr) (accessed on 20 November 2021).

## 3. Results

### 3.1. Colistin Susceptibility Test

Overall, three out of 85 (3.5%) *S*. Infantis strains showed phenotypical resistance to colistin. One strain, isolated from the neck skin of a broiler in 2016 from a slaughterhouse in southern Italy, showed a MIC value = 4 mg/L. The other two strains, isolated from a drumstick in 2017 in a slaughterhouse in northern Italy, presented MIC values = 8 mg/L. The MIC values of the colistin-susceptible isolates ranged from 0.5–1 m/L, and one isolate showed MIC value = 2 mg/L.

### 3.2. Molecular Characterization

Multiplex PCR-screening revealed that the three colistin-resistant *S*. Infantis strains were *mcr*-1-positive.

**Figure 1:**
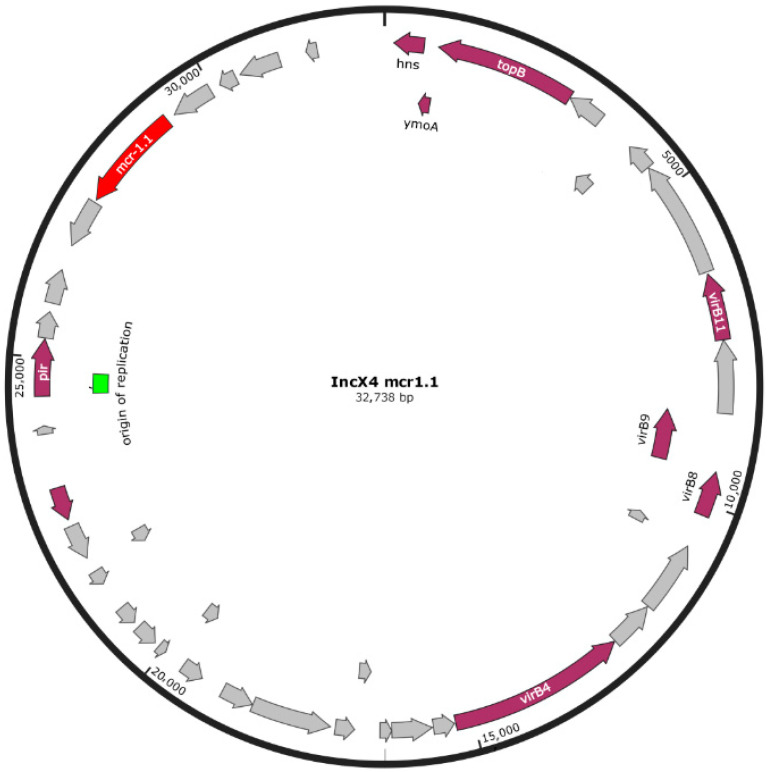
IncX4 plasmid content of the *S*. Infantis isolate, carrying mcr1.1.

**Figure 2:**
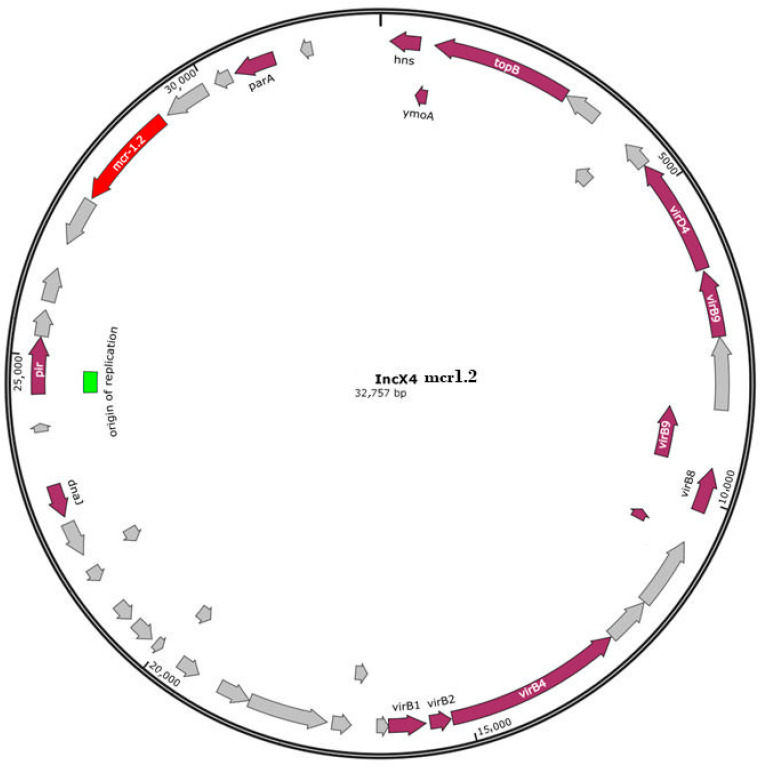
IncX4 plasmid content of the *S*. Infantis isolate, carrying mcr1.2.

The raw data of WGS are shown in Supplementary Material (File S1). The results of the AMR genotype of six *S*. Infantis isolates analyzed by WGS and the phenotypic and PFGE profiles [18] are shown in Table 1. The *mcr*-1.1 variant was detected in one colistin-resistant isolate and *mcr*-1.2 in two colistin-resistant isolates. The two allelic variants showed identical sequences and were located on the IncX4 plasmids in the three *S*. Infantis isolates. (Figure 1 and Figure 2). The isolate harboring the IncX4 plasmid with the *mcr* 1.1 gene also contained the IncFII plasmid.

Resistance to cefotaxime (ESBL phenotype) was predicted by the presence of the *blaCTX-M-1* gene in all cefotaxime-resistant isolates. All isolates were tetracycline-resistant, carrying the tetracycline resistance determinant *tet(A)*. The *dfrA1* determinant mediating trimethoprim resistance was identified in all trimethoprim-resistant isolates [18]; two out of six isolates also harbored the *dfrA14* gene. The *sul1* gene, mediating sulphonamide resistance, was detected in all sulphonamide-resistant strains. All isolates contained *qacE* determinant, mediating quaternary ammonium resistance. We detected pESI-megaplasmid carrying *tet(A)*, *dfrA1*, *dfrA14*, *sul1*, and *qacE* genes in all isolates. The aminoglycoside resistance determinant *aac(6′)-Iaa* was identified in all isolates; one out of six was also gentamicin-resistant, and two out of six aminoglycoside-susceptible isolates harbored *aph(3′*)*-Ia*. Two-point mutations in the quinolone resistance, D→G mutation at position 87 of the *gyrA* gene and T→S mutation at position 57 of the *parC* gene, were detected in all 6 nalidixic acid-resistant and ciprofloxacin-susceptible isolates.

## 4. Discussion

Colistin, considered one of the “last resort” treatments against MDR bacterial infections, may be challenged by the *mcr* genes, which have received widespread attention in different species of *Enterobacteriaceae* found in animals and humans around the world [26]. In this study, we first evaluated, phenotypically and genotypically, the colistin resistance of 85 *S*. Infantis strains and determined the genes conferring AMR by WGS of six *S*. Infantis isolates. Our results demonstrated that three out of 85 (3.5%) strains isolated from slaughtered broilers were colistin-resistant, with MIC values ranging from 4–8 mg/L. In a previous study, Carfora et al. (2018) showed a prevalence of colistin resistance of 1.2% in *S*. Infantis strains from broiler chickens [27]. These data are in agreement with others obtained in Spain [28] and highlight the efficiency of plans, based on the “One Health” approach, applied at UE level and addressed to control the alarming diffusion of antimicrobial resistance in the animal food chain. In EU countries, the use of colistin as a growth promotor has been prohibited since 2006, although its off-label use is permitted in therapeutic treatments with the exception of Finland, Norway and Iceland, where colistin has never been used [29,30]. Following European countries, China has also banned the use of colistin in 2016 [31]. Other Asian countries, such as India, Vietnam and Bangladesh, using this antimicrobial as growth promoter, showed a remarkable prevalence of resistance, with up to 92% of *Salmonella* spp. being colistin-resistant strains in Bangladesh [32]. Additionally, a different colistin susceptibility has been described among *Salmonella* spp. populations. Agerso et al., (2017) demonstrated that *S.* Dublin and *S.* Enteritidis were less susceptible than other *Salmonella* serovars originating from humans [33]. 

The WGS revealed the presence of the *mcr 1.1* and the *mcr-1.2* allelic variants located in the IncX4 plasmids in the three colistin-resistant *S*. Infantis isolates. The two colistin-resistant *S*. Infantis *mcr*-1.2 positive displayed the same XbaI 0126 PFGE profile. The one colistin-resistant isolate *mcr1.1* positive showed the D PFGE profile, a clonally related group to XbaI 0126 [18]. By WGS, the *mcr.1.1* variant was identical to the allelic variant *mcr-1.2*. The same sequence between these two allelic variants was also found by Simoni et al., (2018) in a colistin-resistant blood isolate of *Escherichia coli* [34]. In Italy, the *mcr-1.2* variant, located in the IncX4 plasmid, was first described in *K. pneumonia* isolated from humans [35]. Moreover, this variant, associated with the IncX4 plasmid, was found in *S*. Blockley and in *E. coli* strains isolated from turkeys [36]. To our knowledge, this is the first report of the *mcr1.2* variant described in *S*. Infantis isolated from broiler chickens. We found the *mcr1.1* gene located in the IncX4 plasmid in one colistin-resistant *S*. Infantis isolate, which is in agreement with Carfora et al. [27]. It is known the ability of the *mcr* cassette to jump into several types of plasmids, including the IncX4 plasmid, which seems to be one of the predominant replicon types of plasmids, carrying the *mcr-1* gene [15]. Moreover, the possibility of the transmission of *mcr*-positive IncX4 plasmids among different bacterial species, and from animals to humans or vice versa, has been documented, as reported elsewhere [37]. The WGS revealed that all six isolates carried *blaCTX-M1*, *tet(A)*, *dfrA1* and *sul1* genes, mediating cefotaxime, tetracycline, trimethoprim and sulfonamide resistance, respectively. These genes were located on the pESI-like megaplasmid, as reported by other authors [11,13]. These data are not surprising as, exploiting data from our previous paper [18], the six *S*. Infantis isolates sequenced by WGS were ESBL and resistant toward tetracycline, sulfamethoxazole/trimethoprim and nalidixic acid, thus confirming the typical pattern of multi-resistance of the European *S*. Infantis clone [38]. In *Salmonella* species, DNA gyrase is the primary target of quinolone action; a single-point mutation in the quinolone resistance-determining region (QRDR) of *gyrA* can mediate resistance to nalidixic acid and to ciprofloxacin [39]. In our study, six nalidixic acid-resistant and ciprofloxacin-susceptible *S*. Infantis isolates harbored both *gyrA* and *parC* genes, suggesting that the presence of the two genes makes *Salmonella* strains more susceptible to ciprofloxacin than the isolates harboring a single mutation in *gyrA* [40]. The mutation *parC* is considered rare in salmonellae and was only detected in one out of 382 *S*. Infantis genomes examined in a previous study [13]. Our isolates carried genes, such *parc(C)* and *aph3(III)*, which have not yet been detected in Italian *S*. Infantis strains. The *aph3(III)*, mediating aminoglycoside resistance, was detected in association with *aac(6′)-Iaa* in two aminoglycoside-susceptible *S*. Infantis isolates. Finally, in our study, pESI-megaplasmid also harbored the *qacE* gene, involved in resistance to quaternary ammonium compounds (QACs), extensively used in farm buildings, at the end of the production cycle and in food processing due to its cleaning and disinfectant properties [41]. Jaglic and Cervinkova [42] have already reported that bacteria expressing resistance to antiseptics were generally less susceptible to antibiotic and have hypothesized that outer membrane changes could have played a basic role in this non-specific cross-resistance [43]. More recently, qacH-associated non-classic class 1 integrons were seen in conjugative plasmids that could be a tool responsible for co-dissemination of antimicrobial and disinfectant resistance genes [44].

## 5. Conclusions

The WGS revealed the presence of the *mcr1.1* and *mcr-1.2* allelic variants located in the IncX4-type plasmid. The *mcr-1.2* allelic variant has not yet been described in *S*. Infantis isolated from broiler meat production in Italy. Our results showed a low prevalence of colistin-resistant strains of *S*. Infantis, carrying the *mcr-1* gene, probably due to a reduction in colistin use in poultry [45]. Moreover, the pESI-megaplasmid, detected in our study, in addition to other genes, carried the *qacE* gene involved in resistance to quaternary ammonium salts, one of the most common disinfectants used in the poultry industry. In this work, we confirmed the diffusion and persistence of the ESBL multiresistant S. Infantis strains in broiler meat production chain highlighting the need to improve biosecurity measures both on farms and in slaughterhouses.

## Figures and Tables

**Table 1:** Genotypic and phenotypic profiles of the six *S*. Infantis isolates.

Isolate	Origin	Source	Year	Topology	AMR Genotype	Phenotypic Profile [18]	PFGE [18]
S1	Northern Italy	Neck Skin	2019	Chromosomic	*parC (T57S)*, *blaCTX-M-1*, *aac(6′)-Iaa*, *gyrA(D87G)*	A, Ams, Cl, Ctx, Gm, Na, Sxt, Te	NA
				Plasmidic pESI-like	*sul1*, *qacE*, *dfrA1*, *tet(A)*		
S2	Northern Italy	Neck Skin	2016	Chromosomic	*parC (T57S)*, *blaCTX-M-1*, *aac(6′)-Iaa*, *gyrA(D87G)*	Ams, Cl, Col, Ctx, Caz Na, Sxt, Te	D
				Plasmidic pESI-like	*sul1*, *qacE*, *dfrA1*, *tet(A)*		
				Pasmidic IncX4	*mcr-1.1*		
				Plasmidic IncFII			
S3	Northern Italy	Drumstick	2017	Chromosomic	*parC (T57S)*, *blaCTX-M-1*, *aac(6′)-Iaa*, *aph(3′)-Ia*, *gyrA(D87G)*	A, Cl, Col, Ctx, Na, Sxt, Te	XbaI 0126
				Plasmidic pESI-like	*sul1*, *qacE*, *dfrA1*, *drfA14*, *tet(A)*		
				Plasmidic IncX4	*mcr-1.2*		
S4	Northern Italy	Neck Skin	2017	Chromosomic	*parC (T57S)*, *blaCTX-M-1*, *aac(6′)-Iaa*, *gyrA(D87G)*	A, Ams, Cl, Ctx, Gm, Na, Te	NA
				Plasmidic pESI-like	*sul1*, *qacE*, *dfrA1*, *tet(A)*		
S5	Northern Italy	Drumstick	2017	Chromosomic	*parC (T57S)*, *blaCTX-M-1*, *aac(6′)-Iaa*, *aph(3′)-Ia gyrA(D87G)*	A, Cl, Col, Ctx, Na, Sxt, Te	XbaI 0126
				Plasmidic pESI-like	*sul1*, *qacE*, *dfrA1*, *drfA14*, *tet(A)*		
				Plasmidic IncX4	*mcr-1.2*		
S6	Southern Italy		2016	Chromosomic	*parC (T57S)*, *blaCTX-M-1*, *aac(6′)-Iaa*, *gyrA(D87G)*	A, Ctx, Cz, Na, Sxt, Te	XbaI 0126
				Plasmidic pESI-like	*sul1*, *qacE*, *dfrA1drfA14*, *tet(A)*		

Legend: A (ampicillin), Ams (ampicillin/sulbactam), Cl (cefalexin), Col (colistin), Ctx, (cefotaxime) Caz (ceftazidime), Gm (gentamicin), Na, (nalidixic acid) Sxt (trimethoprim/sulphametoxazole), Te (tetracycline); PFGE (Pulsed field gel electophoresis); NA (profile cluster not assigned); D, (profile cluster not yet labeled by ECDS nomenclature).

## Data Availability

Not applicable.

## Supplementary Materials

The following supporting information can be downloaded at: https://www.mdpi.com/article/10.3390/antibiotics11060728/s1, File S1. WGS data.

## References

[1] Benedict R.G., Langlykke A.F. (1947). Antibiotic Activity of Bacillus Polymyxa. J. Bacteriol..

[2] Poirel L., Jayol A., Nordmann P. (2017). Polymyxins: Antibacterial Activity, Susceptibility Testing, and Resistance Mechanisms Encoded by Plasmids or Chromosomes. Clin. Microbiol. Rev..

[3] Kempf I., Jouy E., Chauvin C. (2016). Colistin Use and Colistin Resistance in Bacteria from Animals. Int. J. Antimicrob. Agents.

[4] Kieffer N., Aires-de-Sousa M., Nordmann P., Poirel L. (2017). High Rate of MCR-1-Producing *Escherichia coli* and *Klebsiella pneumoniae* among Pigs, Portugal. Emerg. Infect. Dis..

[5] Rhouma M., Beaudry F., Thériault W., Letellier A. (2016). Colistin in Pig Production: Chemistry, Mechanism of Antibacterial Action, Microbial Resistance Emergence, and One Health Perspectives. Front. Microbiol..

[6] Kumar H., Chen B.-H., Kuca K., Nepovimova E., Kaushal A., Nagraik R., Bhatia S.K., Dhanjal D.S., Kumar V., Kumar A. (2020). Understanding of Colistin Usage in Food Animals and Available Detection Techniques: A Review. Animals.

[7] Catry B., Cavaleri M., Baptiste K., Grave K., Grein K., Holm A., Jukes H., Liebana E., Lopez Navas A., Mackay D. (2015). Use of Colistin-Containing Products within the European Union and European Economic Area (EU/EEA): Development of Resistance in Animals and Possible Impact on Human and Animal Health. Int. J. Antimicrob. Agents.

[8] World Health Organization (2021). GLASS: The Detection and Reporting of Colistin Resistance.

[9] (2018). European Food Safety Authority; European Centre for Disease Prevention and Control. The European Union Summary Report on Trends and Sources of Zoonoses, Zoonotic Agents and Food-Borne Outbreaks in 2017. EFSA J..

[10] Aviv G., Tsyba K., Steck N., Salmon-Divon M., Cornelius A., Rahav G., Grassl G.A., Gal-Mor O. (2014). A Unique Megaplasmid Contributes to Stress Tolerance and Pathogenicity of an Emergent *Salmonella* Enterica Serovar Infantis Strain. Environ. Microbiol..

[11] Franco A., Leekitcharoenphon P., Feltrin F., Alba P., Cordaro G., Iurescia M., Tolli R., D’Incau M., Staffolani M., Di Giannatale E. (2015). Emergence of a Clonal Lineage of Multidrug-Resistant ESBL-Producing *Salmonella* Infantis Transmitted from Broilers and Broiler Meat to Humans in Italy between 2011 and 2014. PLoS ONE.

[12] Szmolka A., Szabó M., Kiss J., Pászti J., Adrián E., Olasz F., Nagy B. (2018). Molecular Epidemiology of the Endemic Multiresistance Plasmid PSI54/04 of *Salmonella* Infantis in Broiler and Human Population in Hungary. Food Microbiol..

[13] Alba P., Leekitcharoenphon P., Carfora V., Amoruso R., Cordaro G., Di Matteo P., Ianzano A., Iurescia M., Diaconu E.L., Study Group E.-E.-A.N. (2020). Molecular Epidemiology of *Salmonella* Infantis in Europe: Insights into the Success of the Bacterial Host and Its Parasitic PESI-like Megaplasmid. Microb. Genom..

[14] Liu Y.-Y., Wang Y., Walsh T.R., Yi L.-X., Zhang R., Spencer J., Doi Y., Tian G., Dong B., Huang X. (2016). Emergence of Plasmid-Mediated Colistin Resistance Mechanism MCR-1 in Animals and Human Beings in China: A Microbiological and Molecular Biological Study. Lancet Infect. Dis..

[15] Sun J., Fang L.-X., Wu Z., Deng H., Yang R.-S., Li X.-P., Li S.-M., Liao X.-P., Feng Y., Liu Y.-H. (2017). Genetic Analysis of the IncX4 Plasmids: Implications for a Unique Pattern in the Mcr-1 Acquisition. Sci. Rep..

[16] Carattoli A. (2003). Plasmid-Mediated Antimicrobial Resistance in *Salmonella* Enterica. Curr. Issues Mol. Biol..

[17] Yang Q.-E., Sun J., Li L., Deng H., Liu B.-T., Fang L.-X., Liao X.-P., Liu Y.-H. (2015). IncF Plasmid Diversity in Multi-Drug Resistant *Escherichia coli* Strains from Animals in China. Front. Microbiol..

[18] Casagrande Proietti P., Stefanetti V., Musa L., Zicavo A., Dionisi A.M., Bellucci S., Mensa A.L., Menchetti L., Branciari R., Ortenzi R. (2020). Genetic Profiles and Antimicrobial Resistance Patterns of *Salmonella* Infantis Strains Isolated in Italy in the Food Chain of Broiler Meat Production. Antibiotics.

[19] ISO 6579-1:2017/AMD 1:2020 ISO 6579-1:2017/Amd 1:2020. https://www.iso.org/cms/render/live/en/sites/isoorg/contents/data/standard/07/66/76671.html.

[20] Grimont P.A.D., Weill F.-X. (2007). Antigenic Formulae of the Salmonella Serovars.

[21] EUCAST European Committee on Antimicrobial Susceptibility Testing. https://www.eucast.org/fileadmin/src/media/PDFs/EUCAST_files/Breakpoint_tables/v_10.0_Breakpoint.

[22] Rebelo A.R., Bortolaia V., Kjeldgaard J.S., Pedersen S.K., Leekitcharoenphon P., Hansen I.M., Guerra B., Malorny B., Borowiak M., Hammerl J.A. (2018). Multiplex PCR for Detection of Plasmid-Mediated Colistin Resistance Determinants, Mcr-1, Mcr-2, Mcr-3, Mcr-4 and Mcr-5 for Surveillance Purposes. Eurosurveillance.

[23] Bankevich A., Nurk S., Antipov D., Gurevich A.A., Dvorkin M., Kulikov A., Lesin V.M., Nikolenko S.I., Pham S., Prjibelski A.D. (2012). SPAdes: A new genome assembly algorithm and its applications to single-cell sequencing. J. Comput. Biol..

[24] Gurevich A., Saveliev V., Vyahhi N., Tesler G. (2013). QUAST: Quality assessment tool for genome assemblies. Bioinformatics.

[25] Seemann T. (2014). Prokka: Rapid prokaryotic genome annotation. Bioinformatics.

[26] Luo Q., Wang Y., Xiao Y. (2020). Prevalence and Transmission of Mobilized Colistin Resistance (Mcr) Gene in Bacteria Common to Animals and Humans. Biosaf. Health.

[27] Carfora V., Alba P., Leekitcharoenphon P., Ballarò D., Cordaro G., Di Matteo P., Donati V., Ianzano A., Iurescia M., Stravino F. (2018). Corrigendum: Colistin Resistance Mediated by Mcr-1 in ESBL-Producing, Multidrug Resistant *Salmonella* Infantis in Broiler Chicken Industry, Italy (2016–2017). Front. Microbiol..

[28] Vázquez X., García V., Fernández J., Bances M., de Toro M., Ladero V., Rodicio R., Rodicio M.R. (2021). Colistin Resistance in Monophasic Isolates of *Salmonella* Enterica ST34 Collected from Meat-Derived Products in Spain, with or without CMY-2 Co-Production. Front. Microbiol..

[29] Maron D.F., Smith T.J.S., Nachman K.E. (2013). Restrictions on Antimicrobial Use in Food Animal Production: An International Regulatory and Economic Survey. Glob. Health.

[30] Moulin G., Catry B., Baptiste K., Cavaco L., Jukes H., Kluytmans J. (2016). Updated Advice on the Use of Colistin Products in Animals within the European Union: Development of Resistance and Possible Impact on Human and Animal Health. Eur. Med. Agency.

[31] Walsh T.R., Wu Y. (2016). China Bans Colistin as a Feed Additive for Animals. Lancet Infect. Dis..

[32] Uddin M.B., Hossain S.M.B., Hasan M., Alam M.N., Debnath M., Begum R., Roy S., Harun-Al-Rashid A., Chowdhury M.S.R., Rahman M.M. (2021). Multidrug Antimicrobial Resistance and Molecular Detection of Mcr-1 Gene in *Salmonella* Species Isolated from Chicken. Animals.

[33] Agersø Y., Torpdahl M., Zachariasen C., Seyfarth A., Hammerum A.M., Nielsen E.M. (2012). Tentative Colistin Epidemiological Cut-off Value for *Salmonella* spp. Foodborne Pathog. Dis..

[34] Simoni S., Morroni G., Brenciani A., Vincenzi C., Cirioni O., Castelletti S., Varaldo P.E., Giovanetti E., Mingoia M. (2018). Spread of Colistin Resistance Gene Mcr-1 in Italy: Characterization of the Mcr-1.2 Allelic Variant in a Colistin-Resistant Blood Isolate of *Escherichia Coli*. Diagn. Microbiol. Infect. Dis..

[35] Di Pilato V., Arena F., Tascini C., Cannatelli A., Henrici De Angelis L., Fortunato S., Giani T., Menichetti F., Rossolini G.M. (2016). Mcr-1.2, a New Mcr Variant Carried on a Transferable Plasmid from a Colistin-Resistant KPC Carbapenemase-Producing *Klebsiella Pneumoniae* Strain of Sequence Type 512. Antimicrob. Agents Chemother..

[36] Alba P., Leekitcharoenphon P., Franco A., Feltrin F., Ianzano A., Caprioli A., Stravino F., Hendriksen R.S., Bortolaia V., Battisti A. (2018). Molecular Epidemiology of Mcr-Encoded Colistin Resistance in Enterobacteriaceae from Food-Producing Animals in Italy Revealed through the EU Harmonized Antimicrobial Resistance Monitoring. Front. Microbiol..

[37] Viñes J., Cuscó A., Napp S., Alvarez J., Saez-Llorente J.L., Rosàs-Rodoreda M., Francino O., Migura-Garcia L. (2021). Transmission of Similar Mcr-1 Carrying Plasmids among Different *Escherichia coli* Lineages Isolated from Livestock and the Farmer. Antibiotics.

[38] Koutsoumanis K., Allende A., Alvarez-Ordóñez A., Bolton D., Bover-Cid S., Chemaly M., De Cesare A., Herman L., Hilbert F., EFSA Panel on Biological Hazards (EFSA BIOHAZ Panel) (2019). *Salmonella* Control in Poultry Flocks and Its Public Health Impact. EFSA J..

[39] Hopkins K.L., Davies R.H., Threlfall E.J. (2005). Mechanisms of Quinolone Resistance in Escherichia coli and Salmonella: Recent Developments. Int. J. Antimicrob. Agents.

[40] Eaves D.J., Randall L., Gray D.T., Buckley A., Woodward M.J., White A.P., Piddock L.J.V. (2004). Prevalence of Mutations within the Quinolone Resistance-Determining Region of GyrA, GyrB, ParC, and ParE and Association with Antibiotic Resistance in Quinolone-Resistant *Salmonella* Enterica. Antimicrob. Agents Chemother..

[41] Kawai R., Yada S., Yoshimura T. (2019). Characterization and Solution Properties of Quaternary-Ammonium-Salt-Type Amphiphilic Gemini Ionic Liquids. ACS Omega.

[42] Jaglic Z., Cervinkova D. (2012). Genetic Basis of Resistance to Quaternary Ammonium Compounds—The Qac Genes and Their Role: A Review. Vet. Med..

[43] Russell A.D. (2000). Do Biocides Select for Antibiotic Resistance?. J. Pharm. Pharmacol..

[44] Jiang X., Xu Y., Li Y., Zhang K., Liu L., Wang H., Tian J., Ying H., Shi L., Yu T. (2017). Characterization and Horizontal Transfer of QacH-Associated Class 1 Integrons in *Escherichia coli* Isolated from Retail Meats. Int. J. Food Microbiol..

[45] Aconiti Mandolini N., Perugini G., Filippini G., Pierucci P., Baiguini A., Vaccaro A., Pelagalli G., Marinelli F., Capuccella M. (2020). Evaluation of Colistin Consumption in Swine and Poultry of Marche Region during the 2017–2019 Period. Eur. J. Public Health.

